# Experimental Study on the Elastic Support in a Discrete Rail Fastening System Used in Ballastless Tram Track Structures

**DOI:** 10.3390/ma18010141

**Published:** 2025-01-01

**Authors:** Cezary Kraśkiewicz, Monika Urbaniak, Andrzej Piotrowski

**Affiliations:** Faculty of Civil Engineering, Warsaw University of Technology, Al. Armii Ludowej 16, 00-637 Warsaw, Poland; monika.urbaniak.stud@pw.edu.pl (M.U.); andrzej.piotrowski@pw.edu.pl (A.P.)

**Keywords:** ballastless tram track structure, discrete rail fastening system, recycled rubber, baseplate pad

## Abstract

This paper presents an experimental study on the elastic support in a discrete rail fastening system used in a ballastless tram track structure. The study focuses on the elastic support of the anchor element, specifically the Pm49 baseplate. These elements significantly influence environmental pollution along tram routes, such as vibration (at low frequencies) or noise (at high frequencies), as well as static and dynamic rail deflections. The authors outline a methodology for identifying the static and dynamic characteristics of the discrete elastic support in laboratory conditions. The procedure follows the European standard EN 13146-9 for track category A (tramway), as classified according to the European standard EN 13481-5. The study analyzes how the thickness and density of the tested materials affect stiffness. Additionally, it examines the correlation between parameters identified easily on-site (thickness, Shore hardness and density) and laboratory-determined parameters (static and dynamic stiffness), which are costly and time-consuming to measure. The research confirms that prototype prefabricated vibration isolation baseplate pads made of styrene butadiene rubber (SBR) granules, recycled from end-of-life car tires, can achieve equivalent basic static and dynamic parameters, compared to underlays made of two-component polyurethane (PU) resin. This aligns with the strategy of promoting sustainable materials in construction. The innovative and prefabricated SBR rubber baseplate pads can also be used in repair and maintenance works (regardless of weather conditions), as they enable the quick launch of tram traffic. The results of the research included in this article can be used by other scientists, recycled rubber producers, tram track designers or construction site engineers.

## 1. Introduction

The growing population in urban areas is placing considerable pressure on transportation systems, leading to more frequent and severe traffic congestion. One solution to enhance the efficiency of urban transport is to minimize personal vehicle use and promote appealing public transit options, such as an expanded urban rail network [1]. Tram systems offer numerous benefits, including low emissions, which is crucial in light of the rising air pollution from combustion engines. Additionally, tram travel is relatively inexpensive. The use of dedicated tram tracks allows for operations that are largely unaffected by road conditions, making it particularly appealing during peak travel times.

In recent decades, many cities have transitioned from traditional ballasted tram tracks to ballastless alternatives. This change has improved the durability of new routes and enabled the development of “green tracks”, which incorporate soil layers planted with vegetation, thus enhancing urban biodiversity and creating a more pleasant environment for local residents [2,3]. However, ballastless systems [4,5,6,7] come with drawbacks, such as greater rigidity, which can transmit vibrations and noise from passing trams more effectively than ballasted structures. Tram network managers employ various strategies to address this issue, with vibration isolation mats being a common solution.

The research on vibration isolation elements for rail structures has been extensive. For instance, Zhang et al. [8] evaluated the vibration reduction effectiveness of various subway track beds and found that rubber-cushioned floating track beds performed best. Cheng et al. [9] proposed an inverter-enhanced dynamic vibration absorber to improve the mitigation of low-frequency vibration for floating slab tracks. Zhao et al. [10] conducted computational analyses of phononic crystal vibration isolators in floating slab tracks. Xu et al. [11] investigated a slab track with resilient mats and created a model to capture the dynamic properties of these mats. Cai et al. [12] showed, through experiments, that long elastic sleeper tracks used in subways significantly reduced vibrations. Montella et al. [13] examined an innovative floating slab system incorporating rubber-based elastic elements, while Wang et al. [14] introduced a new type of sleeper damping track with elastic side pads, demonstrating similar damping capabilities to floating slab systems. Xu et al. [15] engaged in both theoretical and experimental studies on multi-layer track structures featuring tuned slab dampers.

Typically, resilient vibration isolation materials are made from elastomers like rubber or polyurethane. However, to minimize raw material usage and utilize difficult-to-dispose-of waste, recycled rubber from deconstructed tires has been effectively used in the research on elastic vibration isolators. This approach has also been applied in previous studies, by the authors of this paper, regarding resilient elements made from end-of-life tires.

The authors have primarily focused their research on vibration isolation mats for ballasted track systems, where the use of under ballast mats (UBMs) is standard practice (following the European standard EN 17282 [16]). Additionally, they discussed the impact of mat thickness and density on both the static and dynamic characteristics of mats made from rubber granules and/or fibers [17,18]. Recently, they have also examined resilient elements used in ballastless track systems—under slab mats (USMs) made from end-of-life tires [19]. They introduced methods for the experimental identification and analytical selection of basic static and dynamic parameters of prototype USMs. It should be noted that, so far, the authors have mainly studied UBM/USM mats on railways with a much more porous structure (composed of SBR rubber granulate and fibers), which are located lower in the track structure (under the ballast or track slab) and therefore, the loads are transferred over a larger surface than the prototype baseplate pads analyzed in this article (lower density and based only on SBR rubber granulate), used in the rail fastening system on trams.

Most of the scientific publications to date are based on the existing elastic components of the rail fastening system (baseplate pads, rail pads) from different manufacturers. This article shows that that the authors have developed and tested prototype baseplate pads based on recycled rubber (SBR rubber granulate), proving that they can be an equivalent product (taking into account the basic static and dynamic parameters) to the standardly used materials from two-component PU resins.

In this study, the authors present the results of laboratory tests aimed at identifying the basic static and dynamic parameters, and evaluating the correlation between the hardness and density, of the vibration isolation pad material—the resilient support of the Pm49 baseplate made of SBR (styrene-butadiene rubber) granules and two-component polyurethane resin—and its static and dynamic stiffness. The main objective of this work is to prove that the prefabricated SBR-based pads can be a viable alternative to underlays made of two-component polyurethane resins, and that their static and dynamic properties can be tailored to the requirements of a given project.

## 2. Discrete Rail Fastening System with a Baseplate

This section presents a discrete fastening system of the Vignole rail with a 49E1 profile [20], with a particular emphasis on the location of the vibration isolation pad element under study. Vibration isolation pads are the key element that acts as an elastic support for the Pm49 baseplate in the rail fastening system. Their proper operation reduces vibration and noise and increases the durability of the entire track superstructure as a result of its increased resilience.

Traditional polyurethane resin underlays require time-consuming installation under strict technological regimes. The process begins with a thorough cleaning and priming of the surfaces (the concrete track slab and the underside of the steel baseplate) that will come into contact with the resin from dust, oil and other contaminants. The formwork is then installed and carefully sealed to prevent leakage of the liquid resin. The prepared and vented mixed resin is then poured into the mold and evenly distributed. After a few days, when the resin has completely hardened, the mold is removed. Quality control is then carried out, and if necessary, the surface can be lightly polished. Rubber baseplate pads, on the other hand, due to their prefabricated nature, are assembled directly and do not require suitable weather conditions.

The production process of baseplate pads based on recycled rubber (SBR) begins with shredding rubber from used car tires into rubber granules. The rubber granules are then bonded with polyurethane glue in a hydraulic press under high pressure into a prefabricated baseplate pad. Such pads are delivered to the construction site as prefabricated pieces with their dimensions (length and width) adapted to the dimensions of the Pm49 steel baseplate (i.e., 345 × 160 mm). Using the thickness and/or density of the pad, its static and dynamic stiffness can be controlled, as detailed later in the article.

**Figure 1:**
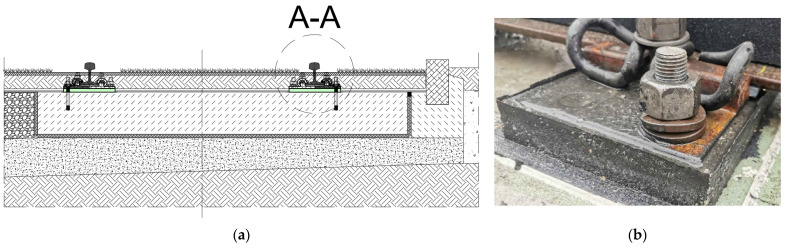
Ballastless green tram track structure of the Vignole rail 49E1 with a discrete fastening system: (**a**) cross-section; and (**b**) photo of the discrete rail fastening system with Pm49 baseplate supported by the two-component PU resin-based underlay.

**Figure 2:**
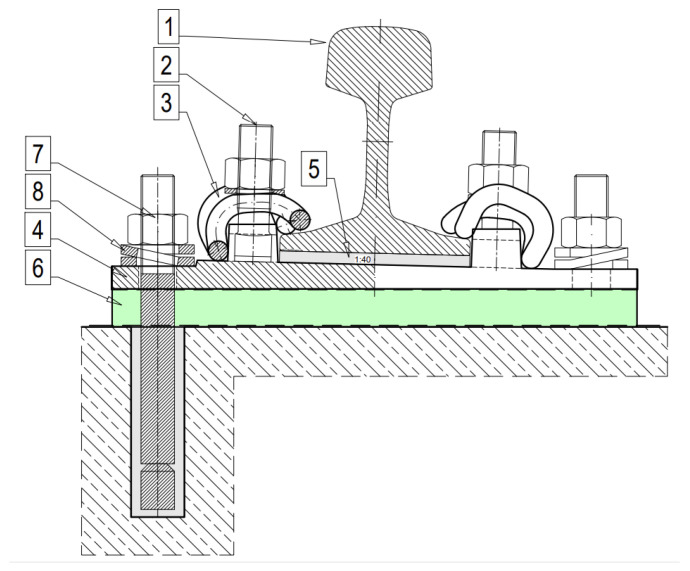
Details of the discrete rail fastening system with a baseplate Pm49 standard support using the two-component PU resin-based underlay. Symbols: 1—Vignole rail with a 49E1 profile; 2—alloy T-bolt and nut with washer; 3—elastic rail clip/tension clamp type Skl 12; 4—baseplate type Pm49; 5—shaped rail pad type PAK; 6—vibration isolation/baseplate pad (made of SBR granules—recycling material or two-component PU resin-based material), 7—steel anchors glued with epoxy glue into the concrete slab; and 8—double-coil spring rings.

**Figure 3:**
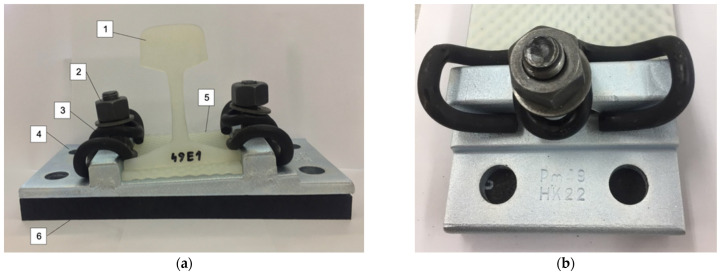
Scheme of the discrete resilient rail fastening system for ballastless tram tracks: (**a**) View of the whole system; and (**b**) close-up with visible Pm49 baseplate. Symbols: 1—Vignole rail with a 49E1 profile; 2—alloy T-bolt and nut with washer; 3—elastic rail clip/tension clamp type Skl 12; 4—baseplate type Pm49; 5—shaped rail pad type PAK; 6—vibration isolation/baseplate pad (made of SBR granules—recycling material).

Figure 1 depicts a sample cross-section of the ballastless green tram track structure and a photo from the construction site of the standard variant with a Pm49 baseplate supported by the two-component PU resin-based underlay. The details of the discrete rail fastening system are presented in Figure 2 and Figure 3. The SBR baseplate pads are compressed by prestressing the steel anchors, glued with epoxy glue, into the concrete slab. To prestress the anchors, the nuts on the anchors are tightened to a set torque, and double-coil spring rings generate the appropriate compression force.

The static and dynamic loads generated by the vehicle are first transferred to the rail pad (Figure 3), which partially absorbs the impacts. Then, the loads are transferred through the elastic footings to the baseplate, and from there to the vibration isolation pad, which further dampens vibrations and provides an elastic support to the entire rail fastening system. Finally, the load is transferred through the track slab to the track substructure, and further into the soil medium.

Therefore, the continuous research and optimization of vibration isolation materials are essential to improve the effectiveness of vibration damping and minimize its impact on urban infrastructure. In addition, the analysis of such systems allows for a better understanding of the behavior of track structures under static and dynamic loads, which is crucial for the further development of materials engineering in this field [21].

## 3. Laboratory Tests

### 3.1. Resilient Support Samples and Test Stand

Laboratory tests on material samples that provide elastic support for a baseplate were carried out in the Laboratory of the Faculty of Civil Engineering at the Warsaw University of Technology. The tests covered both the basic characteristics of each specimen, such as dimensions, mass, density, and Shore hardness, as well as more advanced ones, such as static and dynamic stiffness. The dynamic elastic characteristics were determined at low (force control method) and high (displacement control method) frequencies.

**Figure 4:**
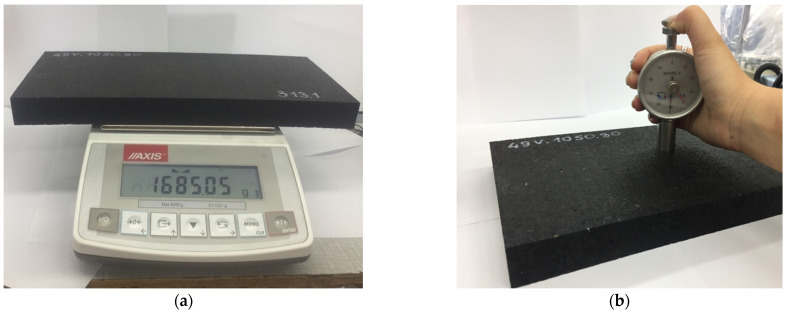
Basic tests on resilient SBR-based pads: (**a**) determination of mass; and (**b**) determination of Shore hardness.

**Figure 5:**
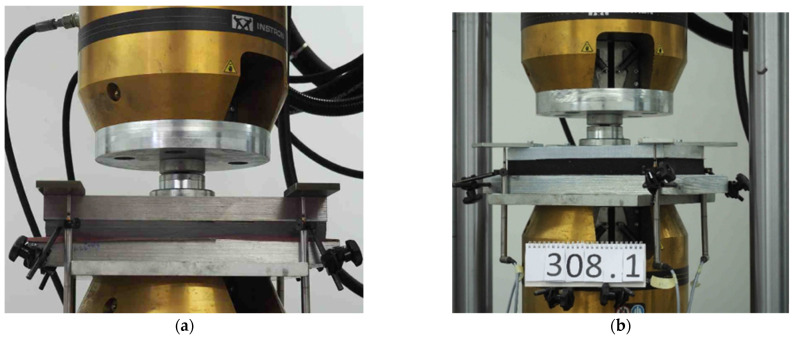
Test stand for determination of static and dynamic elastic characteristics of discrete elastic supports of the baseplate/rail: (**a**) polyurethane-based specimen, 25 mm thick; and (**b**) SBR-based specimen, 30 mm thick.

**Figure 6:**
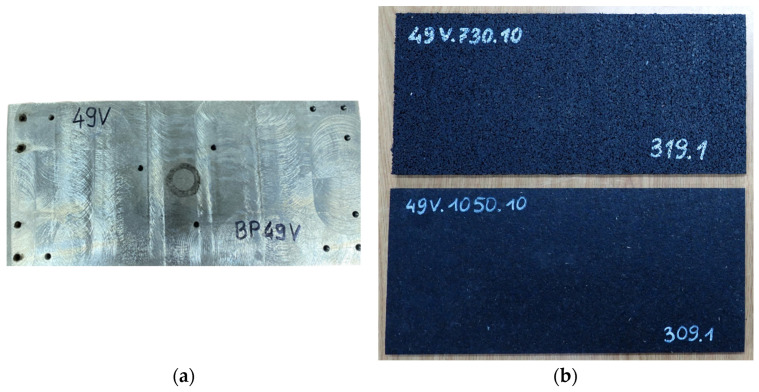
Load distribution plate and baseplate pads of 345 mm × 160 mm used in static and dynamic stiffness tests: (**a**): plate simulating the pressure of the Pm49 baseplate; and (**b**) baseplate pads of two different densities, 0.73 g/cm^3^ (upper photo) and 1.05 g/cm^3^ (lower photo).

The examples of SBR-based specimens under basic tests are shown in Figure 4, and the test stand for the determination of the static and dynamic elastic characteristics of the pads is presented in Figure 5. Figure 6 depicts a load distribution plate used in the tests.

Laboratory tests were carried out according to uniform standard procedures, taking into account the fact that the resilient support, despite the different type of material used (rubber granules—SBR or polyurethane resin—PU) and thus different assembly technology, perform the same function of being a discrete resilient support of the Pm49 baseplate and the Vignole rail with a 49E1 profile.

To make it easier to identify successive specimens, a system of specimen designations that includes key information about the material, thickness and density of the specimen has been introduced. For example, the designation: “307_SBR_25_1.10” indicates that specimen No. 307 was made of SBR granules, has a nominal thickness of 25 mm and a nominal density of 1.10 g/cm^3^, while samples labeled “PU” were made of two-component polyurethane resin.

The research covered a wide range of samples with different thicknesses and densities. A summary of selected properties of all tested samples is presented in Table 1, where *k_SP_* is a static stiffness, *k_LFPf_* is a dynamic stiffness at low frequencies, and *k_HFPf_* is a dynamic stiffness at high frequencies, determined according to EN 13146-9 [22] and EN ISO 10846-2 [23] for track category A (tramway), classified according to the European standard EN 13481-5 [24].

### 3.2. Methodology

This study employed a systematic methodology to evaluate the suitability of vibration isolation pads made from recycled SBR granules as an alternative to conventional polyurethane (PU) resin underlays. The materials used included prefabricated baseplate pads from SBR granules, which were derived from end-of-life car tires, and PU-based two-component resin underlays, which served as the reference material due to their established use in ballastless tram tracks. The SBR samples were prepared in a range of nominal densities from 0.73 g/cm^3^ to 1.10 g/cm^3^, with thicknesses varying between 10 mm and 30 mm, to represent the diverse properties needed for practical applications. Each material’s Shore hardness was measured using standard durometers (type: A, A0 and D), and their densities were verified to ensure consistency with the specified design.

The research was conducted in distinct phases. First, the basic physical characteristics of the samples, including the dimensions, mass (Figure 4a), density, and Shore hardness (Figure 4b), were measured to establish a baseline for comparison. Next, the elastic properties of the baseplate pads were evaluated through static and dynamic stiffness tests (Figure 5) conducted in accordance with European standards, including EN 13146-9 [22] and EN ISO 10846-2 [23]. The static stiffness *k_SP_* was measured using a universal testing machine under controlled load conditions (Table 2 and Table 3), while the dynamic stiffness was assessed at both low (1–20 Hz; Table 4) and high (8–24 Hz; Table 5) frequencies. The low frequency dynamic stiffness *k_LFPf_* (force-controlled method) is used to determine the dynamic deflections of the rail/baseplate, while higher frequency dynamic stiffness *k_HFPf_* (displacement-controlled method) is used to analyze the reduction in the vibration level from the tram route to its surroundings (environment and nearby buildings).

Therefore, a statistical correlation analysis was performed to explore the relationships between the material properties—such as density, thickness, and hardness—and their corresponding stiffness values. This analysis aimed to identify trends and establish predictive correlations that could simplify the selection of materials for future applications. Finally, an equivalence assessment was carried out to compare the performance of the SBR-based baseplate pads against the PU-based underlays. The equivalence was determined based on key indicators, including static and dynamic stiffness values.

All experiments were conducted in the Laboratory of the Faculty of Civil Engineering at the Warsaw University of Technology. The experimental setup included specialized equipment, such as a universal testing machine Instron 8802 (Figure 5) and custom-designed load distribution plate (Figure 6a), to simulate the pressure of the Pm49 baseplate. By combining rigorous laboratory testing with detailed data analysis, this study provides a comprehensive evaluation of SBR-based baseplate pads as a sustainable alternative to PU resin underlays in vibration isolation systems of ballastless tram track structures.

### 3.3. Results of Static and Dynamic Tests

#### 3.3.1. Influence of the Pad Thickness on Its Static and Dynamic Properties

This section discusses the effects of selected parameters, such as the thickness and density of the specimen, on the static and dynamic stiffness of the elastic support of the baseplate. The applied approach makes it possible to draw conclusions as to whether rubber granule pads can exhibit similar static and dynamic properties to resin underlays at the same thickness (usually derived from design assumptions) and whether they can be used in the design of sustainable track structures including, for example, repair programs and maintenance works. Due to the large number of results obtained, this section mainly focuses on the analysis of five SBR-based samples with a nominal density of 0.73 g/cm^3^ and four PU-based samples: three with a nominal density of 1.00 g/cm^3^ and one with a nominal density of 1.15 g/cm^3^.

**Figure 7:**
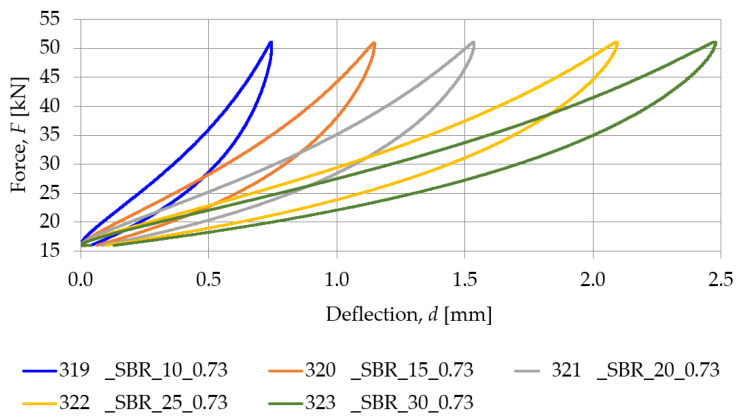
Static elastic characteristics of SBR-based samples with a nominal density of 0.73 g/cm^3^.

**Figure 8:**
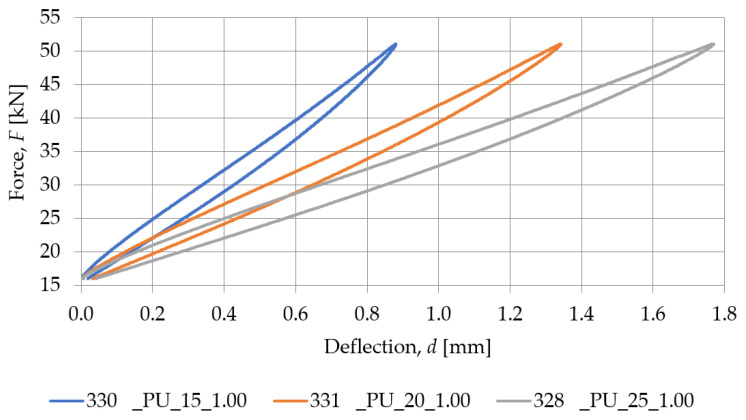
Static elastic characteristics of PU-based samples with a nominal density of 1.00 g/cm^3^.

Figure 7 presents a comparison between the SBR-based samples with a similar density (nominal value of 0.73 g/cm^3^) and different nominal thicknesses (10, 15, 20, 25, and 30 mm), while the static stiffness values determined for these samples are gathered in Table 2. Similarly, the comparison between PU-based samples with a nominal density of 1.00 g/cm^3^ and differing thicknesses (15, 20, and 25 mm) is shown in Figure 8, and the static stiffness values are presented in Table 3.

It can be noticed that both SBR- and PU-based samples show the same tendency in terms of thickness–static stiffness relationship. Specimens with a greater thickness show higher deflection at the same load, resulting in lower stiffness compared to specimens with a lower thickness, which exhibit lower deflection at similar force values.

**Figure 9:**
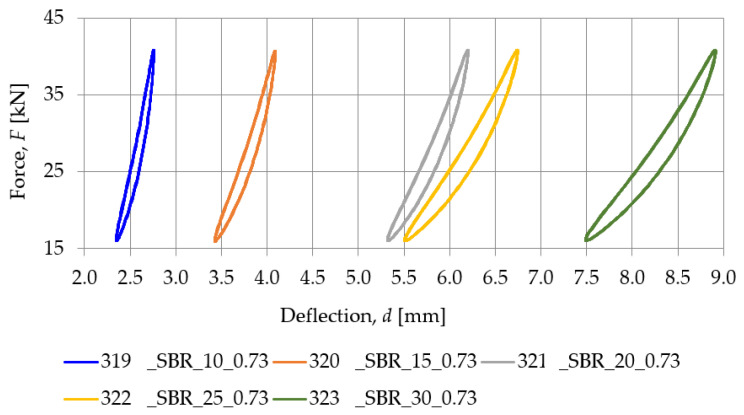
Dynamic elastic characteristics, at 5 Hz, of SBR-based samples with a nominal density of 0.73 g/cm^3^.

**Figure 10:**
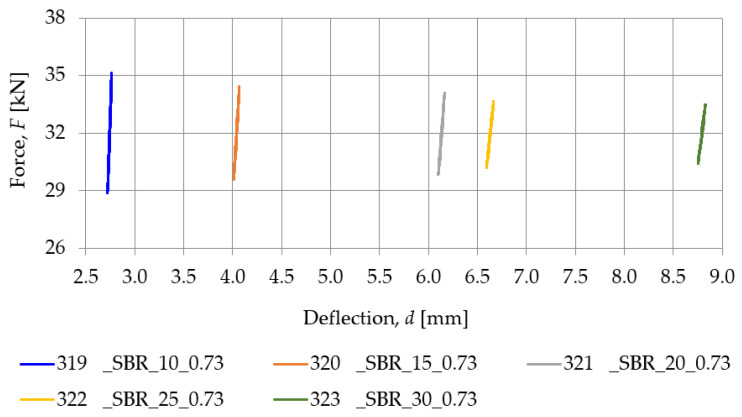
Dynamic elastic characteristics, at 20 Hz, of SBR-based samples with a nominal density of 0.73 g/cm^3^.

Figure 9 and Table 4 show the typical behavior of SBR-based samples under a dynamic load at low frequencies. Similarly, Figure 10 and Table 5 are concerned with the same SBR-based samples, but under the dynamic tests performed at high frequencies. The dynamic stiffness at low frequencies decreases as the thickness of the specimens increases, implying that the pads of smaller thickness exhibit lower values of dynamic deflections from passing tram vehicles and lower vibration isolation level.

**Figure 11:**
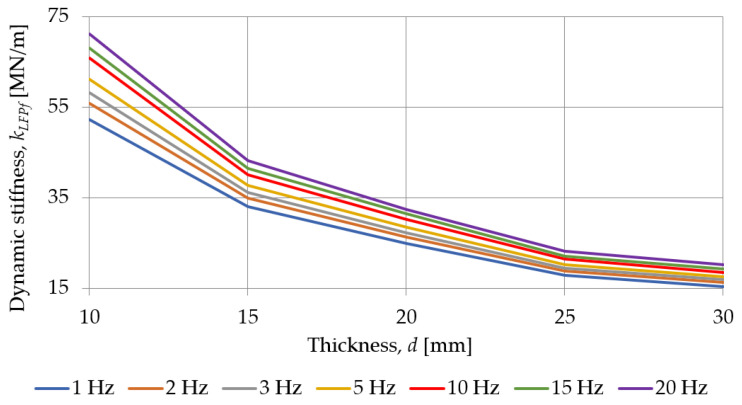
Influence of the pad thickness on its dynamic stiffness at low frequencies determined for SBR-based samples with a nominal density of 0.73 g/cm^3^.

**Figure 12:**
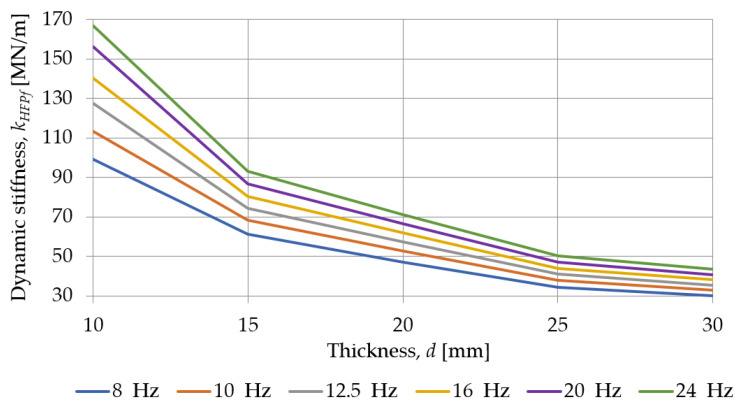
Influence of the pad thickness on its dynamic stiffness at high frequencies determined for SBR-based samples with a nominal density of 0.73 g/cm^3^.

Figure 11 and Figure 12 show how the dynamic stiffness, at low and high frequencies, determined for SBR-based samples with a nominal density of 0.73 g/cm^3^, depends on their thickness. For all test frequencies, the dynamic stiffness of the pad decreases with an increase in its thickness.

**Figure 13:**
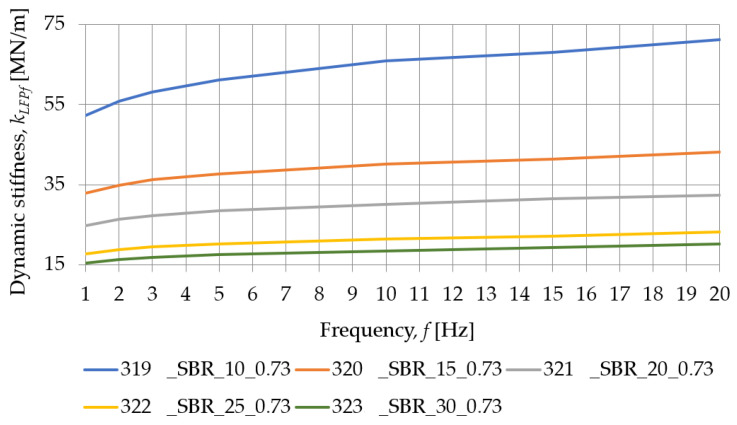
Influence of the load frequency on the dynamic stiffness at low frequencies determined for SBR-based samples with a nominal density of 0.73 g/cm^3^.

**Figure 14:**
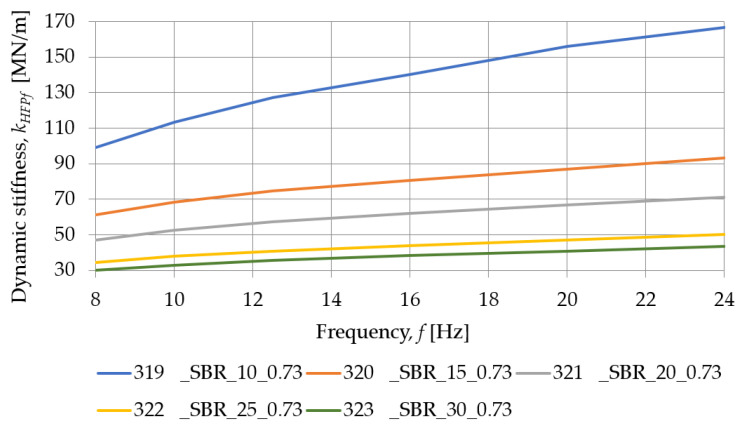
Influence of the load frequency on the dynamic stiffness at high frequencies determined for SBR-based samples with a nominal density of 0.73 g/cm^3^.

The influence of the load frequency on the dynamic stiffness of SBR-based samples with a nominal density of 0.73 g/cm^3^ is presented in Figure 13 and Figure 14. As the thickness of the pad increases, the dynamic stiffness decreases, and the smaller it’s thickness, the greater the effect of the frequency of the test on the stiffness of the sample.

An analysis of the results presented above indicates that both the static and dynamic stiffnesses decrease as the thickness of the pad increases, regardless of its material. This is consistent with the theoretical results, where the greater thickness of the element leads to a higher deformability under the same load.

For specimens made of PU resin, with a similar volumetric density (~0.99 g/cm^3^), differences in thickness significantly affect stiffness values. Similarly, for SBR specimens with similar nominal densities but different thicknesses, different stiffnesses are observed, suggesting that thickness has a significant effect on static and dynamic stiffness values. There is an inverse relationship between the thickness of the pad and its static and dynamic stiffness. Regardless of the type of material, an increase in specimen thickness always leads to a decrease in the stiffness of the elastic support of the baseplate.

#### 3.3.2. Influence of the Pad Density and Material on Its Static and Dynamic Properties

**Figure 15:**
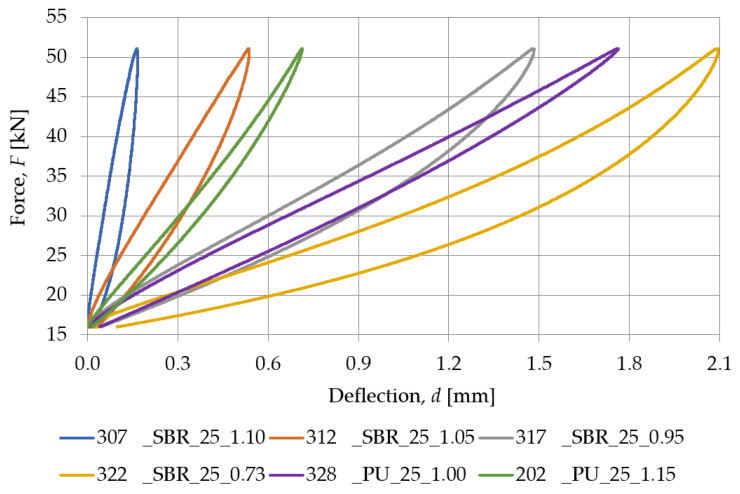
Static elastic characteristics of SBR- and PU-based samples with a nominal thickness of 25 mm.

Figure 15 and Table 6 show the static characteristics of samples with different densities but a similar nominal thickness. As the SBR material density increases, the static stiffness grows. These results suggest that higher-density SBR materials exhibit greater resistance to deformation under static loading.

**Figure 16:**
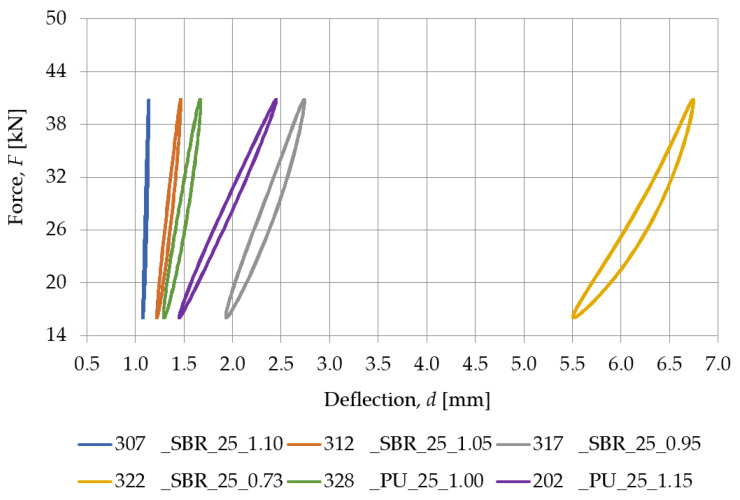
Dynamic elastic characteristics, at 5 Hz, of SBR- and PU-based samples with a nominal thickness of 25 mm.

Figure 16 shows the dynamic elastic characteristics determined for samples of different densities and hardnesses, but similar nominal thickness, at 5 Hz. It can be noticed that the dynamic stiffness of SBR-based pads increases with increasing material density, which suggests that higher-density SBR-based materials exhibit greater resistance to deformation under static loading.

**Figure 17:**
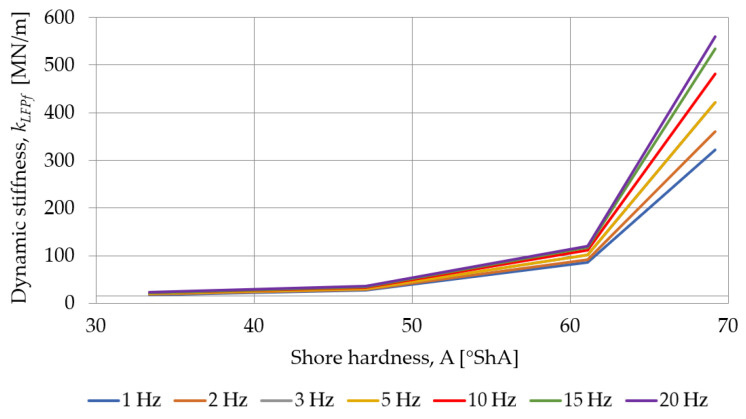
Influence of the Shore hardness A on the dynamic stiffness at low frequencies determined for SBR- and PU-based samples with a nominal thickness of 25 mm.

**Figure 18:**
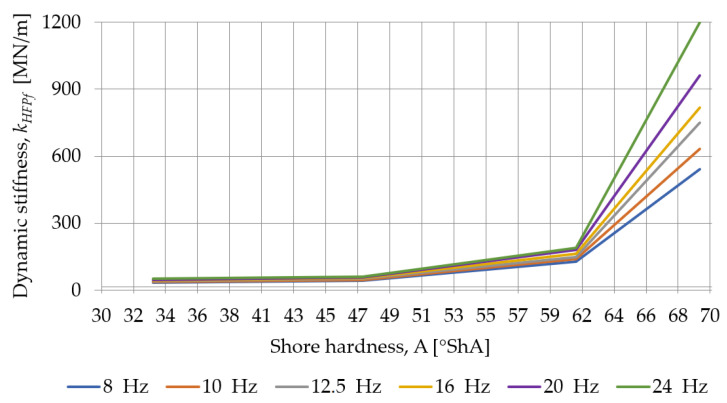
Influence of the Shore hardness A on the dynamic stiffness at high frequencies determined for SBR- and PU-based samples with a nominal thickness of 25 mm.

The influence of the Shore hardness A on the dynamic stiffness, at low and high frequencies, determined for SBR- and PU-based samples with a nominal thickness of 25 mm, is presented in Figure 17 and Figure 18. As the hardness of the pad increases, the dynamic stiffness increases, and the greater the hardness, the greater the effect of the frequency of the test on the stiffness of the sample.

**Figure 19:**
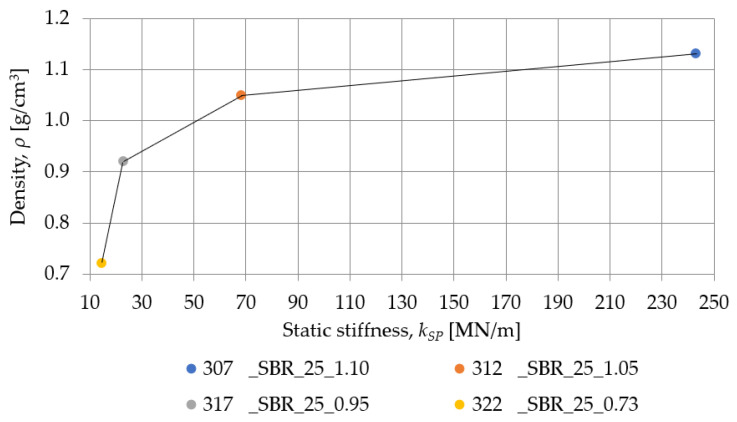
Influence of the density on the static stiffness of SBR- and PU-based samples with a nominal thickness of 25 mm.

The influence of the density on the static stiffness of SBR- and PU-based samples with a nominal thickness of 25 mm is shown in Figure 19. In materials characterized by the same thickness, the value of static stiffness depends on its density and hardness, but the effect of the material hardness on static stiffness occurs only if the difference in hardness between materials is significant. The greater the hardness, the greater the static and dynamic stiffness of a given specimen. This is one of the properties that allows for the faster initial identification/verification of materials. At the same time, it can be noted that density and hardness affect the values of static stiffness logarithmically, especially in the case of samples made of SBR granules. Thus, it should be taken into account that, with an increase in the density and hardness of SBR-based pads, there is a significant increase in stiffness, and for this reason, special attention is recommended when designing the density of such pads.

The analysis of the results presented above clearly shows that material density has a significant effect on both static and dynamic elastic characteristics. Increasing the material density of vibration isolation pads leads to an increase in both static and dynamic stiffness.

### 3.4. Correlation Analysis

The correlation coefficient is a parameter used to determine the relationship between two sets of data (variables). It also allows you to identify the type of relationship. The value of the correlation coefficient ranges from −1 to 1. The sign of the coefficient is important: a positive sign indicates that an increase in one variable is associated with an increase in the other, while a negative sign means that an increase in one variable causes a decrease in the other. The subsets determining the correlation strength are presented in Table 7.

The correlation coefficient is calculated as:

(1) $$r_{xy}=\frac{\sum_{i=1}^{n}\left(x_{i}-\bar{x}\right)\cdot (y_{i}-\bar{y})}{\sqrt{\sum_{i=1}^{n}{\left(x_{i}-\bar{x}\right)}^{2}}\cdot \sqrt{\sum_{i=1}^{n}{\left(y_{i}-\bar{y}\right)}^{2}}},$$

where $x_{i}$ and $y_{i}$ are the variables from the first and second set, respectively, and $\bar{x}$ and $\bar{y}$ are arithmetic means of the first and second set, respectively.

The correlation analysis of selected properties of vibration isolation pads aims to identify relationships between individual parameters that can affect their effectiveness, for example, in vibration isolation or static and dynamic deflections of rails or baseplates. Understanding the relationships between material properties can lead to simplified testing and manufacturing processes, eliminating the need for extensive laboratory testing when some properties can be predicted or pre-verified from other properties.

**Figure 20:**
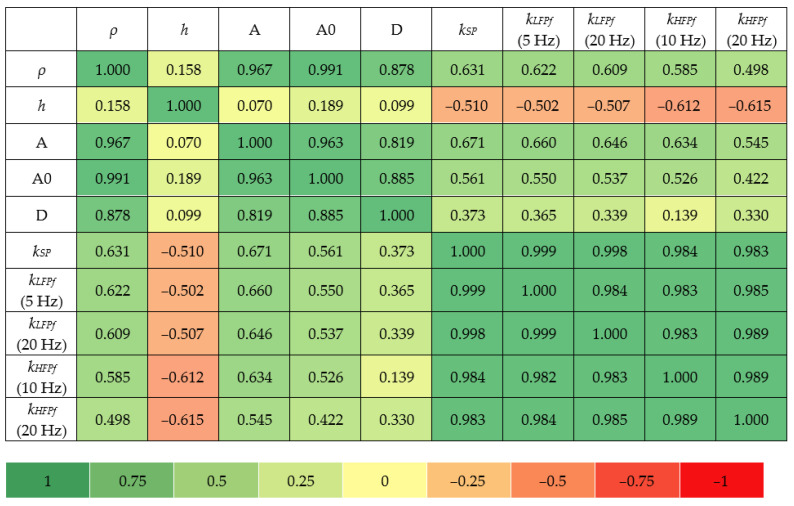
Correlation matrix for SBR-based samples.

The correlation matrix in Figure 20 shows a visual representation of the relationship between various properties of the tested materials—including density, thickness, Shore hardness (type A, A0, D), static stiffness and dynamic stiffness at low frequencies (5 and 20 Hz) and high frequencies (10 and 20 Hz)—for pads made of SBR granules. In the correlation analysis, 11 types of SBR-based samples with nominal densities in the range of 0.73–1.05 g/cm^3^ were considered. Samples with nominal densities of 1.10 g/cm^3^ were omitted due to the significant scatter of results.

Based on the correlation matrix (Figure 20), the following conclusions can be drawn:

- A strong positive correlation (min. 0.878) of density with Shore hardness (A, A0, D), indicates that materials with higher densities have higher Shore hardness values.
- A moderate positive correlation of density with static stiffness (0.631), dynamic stiffness (force method) at 5 Hz (0.622) and 20 Hz (0.609), and dynamic stiffness (displacement method) at 10 Hz (0.585) and 20 Hz (0.498), indicates that materials with higher densities tend to have higher stiffness.
- A weak positive correlation (less than 0.189) of thickness with hardness at different Shore scales, means that thickness can only affect the hardness of the sample slightly.
- Moderate negative correlations of thickness with static stiffness (−0.510), dynamic stiffness (force method) at 5 Hz (−0.502) and 20 Hz (−0.507), and dynamic stiffness (displacement method) at 10 Hz (−0.612) and 20 Hz (−0.615) suggest that thicker materials tend to exhibit lower stiffness.
- Strong positive correlations (min. 0.819) between different Shore hardness scales indicate a high level of consistency in Shore hardness measurements evaluated at different scales—i.e., tested with different durometers (types A, A0 and D).
- Moderate positive correlations of A-type and A0-type Shore hardness with static stiffness (min. 0.561), dynamic stiffness (force method) at 5 Hz (min. 0.550) and 20 Hz (min. 0.537), and dynamic stiffness (displacement method) at 10 Hz (min. 0.526) and 20 Hz (min. 0.422) suggest that harder materials tend to be stiffer.
- Weak positive correlations of D-type Shore hardness with static stiffness were observed (0.139; 0.373); therefore, it is suggested that an A- or A0-type durometer be used when testing hardness on-site, due to the stronger correlations with stiffness.

**Figure 21:**
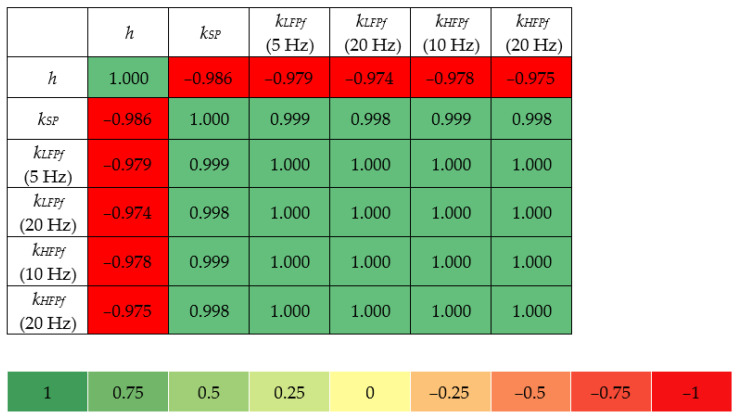
Correlation matrix for PU-based samples.

Similarly, the correlation matrix in Figure 21 shows a visual representation of the relationship between various properties of the tested materials for pads made of PU resin. Due to the small number of PU-based samples with similar nominal densities, the range of properties analyzed in the correlation matrix was limited, focusing on the analysis of the correlation between thickness and static and dynamic stiffness.

Based on the correlation matrix (Figure 21), it can be concluded that the strong negative correlation of thickness with static stiffness (−0.986) means that thicker materials show lower stiffness. The same trend was observed for dynamic stiffness (force method) at 5 Hz (−0.979) and 20 Hz (−0.974), and dynamic stiffness (displacement method) at 10 Hz (−0.978) and 20 Hz (−0.978), which proves that thicker samples have lower stiffness.

### 3.5. Equivalence Assessment of SBR-Based Pads Against PU-Based Underlays

Based on the results presented in previous sections, particularly with regard to stiffness and Shore hardness, a proposal for equivalent materials based on recycled rubber relative to resilient polyurethane resin underlays was prepared. The details of the equivalent materials are shown in Table 8.

An analysis of the properties of tested samples leads to the conclusion that the unambiguous determination of the trend in the selection of equivalent materials from SBR granules relative to PU resin underlays is a complex issue and requires an iterative approach. However, it should be noted that the stiffness is the result of the tangent of the angle of inclination of the elastic characteristic with respect to the horizontal axis, so the equivalent material can be selected mainly based on the numerical values of stiffness, without the need for a detailed analysis of the graph of static and dynamic characteristics. It is also worth noting that for PU-based samples No. 328 and 331, the same equivalent was selected from SBR-based pads (No. 317 with a nominal density of 0.95 g/cm^3^) due to the low availability of samples with a nominal density of 1.05 g/cm^3^. Nevertheless, it can be indicated that for PU-based samples, equivalent materials of similar nominal thickness should be selected from SBR-based pads with a nominal density in the range of 0.95–1.05 g/cm^3^. Such a range is an important convenience in the iterative process of selecting equivalent SBR-based samples.

Prefabricated SBR-based pads can be a viable alternative to PU-based underlays in applications where vibration damping capability is key and where material resilience is required. There are pads made of SBR granules with similar properties to PU-based samples, although it is impossible to find a clear trend in the translation of the two materials. In order to find an SBR-based pad that will be equivalent to a PU-based one, SBR materials with a density in the range of 0.90–1.05 g/cm^3^ should be considered. This is due to the logarithmic dependence of hardness and density on the stiffness of the pad, where once the Shore A hardness exceeds 65, the stiffness of the sample begins to increase rapidly.

Similar trends were observed for rubber samples with a nominal density of above 1.10 g/cm^3^. However, it is worth noting that both the density and hardness of SBR-based samples have a very strong correlation with each other (min. 0.878), so it can be concluded that when one of these parameters increases, one can expect an increase in all three to a similar degree. At the same time, after observing a very moderate negative correlation between thickness and static and dynamic stiffness (−0.510; −0.615), it can be concluded that, despite the sharp increase in static and dynamic stiffness in the case of increased density and hardness, it is possible to slightly correct the stiffness by using thicker SBR-based pads. However, such a correction can only be applied to a limited extent due to the increase in density and hardness with increased thickness (correlation 0.070–0.189).

SBR granulate is a cheaper material to produce compared to PU resin. In addition, the ease and speed of installation of prefabricated SBR-based pads can contribute to lower track construction costs. SBR granules may be a viable alternative in terms of the resilient support of the baseplate with respect to PU resin in applications where vibration damping capability is important (related to dynamic characteristics) and where a certain elasticity of the material is required to condition the values of maximum deflections of the rail/baseplate (related to static and dynamic characteristics).

## 4. Conclusions

In this paper, an experimental study on the elastic support of a discrete rail fastening system in a ballastless tram track structure was presented. Selected properties of the resilient baseplate pads, made of SBR granules, and underlays, made of PU-based two-component resin, were identified, with the main purpose of evaluating their suitability for use in the construction of tram track systems.

The feasibility of developing SBR-based baseplate pads with equivalent properties to PU-based underlays was confirmed through the proper selection of rubber baseplate pad parameters, such as density and hardness, and pad thickness. A prefabricated rubber baseplate pad with the right parameters can effectively combine the damping properties of rubber with the required static and dynamic stiffness, offering sustainable solutions for tram tracks. The use of recycled tire rubber baseplate pads, due to the lower cost of raw materials (SBR granules) used in their production, can lead to significant savings on the part of the investor and contractor for large engineering projects. The possibility of recycling end-of-life car tires and using them in the production of resilient baseplate pads promotes the sustainable development of rail transportation and reduces its negative impact on the environment.

The advantage of baseplate pads made from SBR rubber granulate over those made from traditional PU resins also results from the use of prefabricated elements with elastic characteristics tested in the production plant (as part of factory production control) and the possibility of installing the rail fastening system in difficult weather conditions (two-component PU resins have high requirements regarding temperature and humidity ranges enabling correct application on the construction site). An additional advantage is the possibility of starting tram traffic immediately after compressing/installing the rail fastening system (PU resins only reach their target hardness and stiffness after several weeks).

The authors have participated in the development of tram track repair programs many times—including those installed in bad weather conditions (e.g., application of two-component PU resin at too low temperatures). The innovative and prefabricated SBR rubber baseplate pads can also be used in repair and maintenance works (regardless of weather conditions), as they enable the quick launch of tram traffic.

The choice between the two types of materials should be made on the basis of a detailed analysis of the specific requirements of a given project, taking into account both the technical and economic aspects. Ultimately, SBR-based pads, thanks to their versatile properties and the possibility of selection based on parameter correlation, can find wide application in modern tram track vibration isolation systems, offering effective and economical solutions.

The results of the research included in this article can be used by other scientists, recycled rubber producers, tram track designers or construction site engineers (initial material control), so the article has universal significance (scientific and application).

In further research, the authors plan to consider the electric conductivity properties of PU and SBR, as they are very important factors that should be taken into account while implementing new materials into a fastening system structure. Urban railway systems are often exposed to high levels of stray currents and related corrosion. The differences in material composition can change the electrical insulation of the rail to supporting structures, leading to potential current drain and the corrosion of rail and fastening system elements [26,27].

## Figures and Tables

**Table 1:** Selected properties of tested samples.

Sample No.	Density *ρ* [g/cm^3^]	Thickness *h* [mm]	Shore Hardness A [°ShA]	Shore Hardness A0 [°ShA0]	Shore Hardness D [°ShD]	Stiffness *k_SP_* [MN/m]	Stiffness *k_LFPf_* (5 Hz) [MN/m]	Stiffness *k_LFPf_* (20 Hz) [MN/m]	Stiffness *k_HFPf_* (10 Hz) [MN/m]	Stiffness *k_HFPf_* (20 Hz) [MN/m]	Material
307	1.13	25.21	68.9	80.6	20.4	233.860	409.274	543.246	596.648	936.956	SBR
308	1.08	29.86	65.1	79.1	19.0	72.299	109.485	131.200	146.601	185.400	SBR
312	1.05	24.83	61.3	77.1	17.8	70.601	105.716	125.463	144.021	184.431	SBR
313	1.04	29.71	60.8	77.4	17.9	41.905	60.586	70.916	83.456	103.292	SBR
314	0.99	10.27	59.1	73.1	15.4	103.886	157.961	189.603	215.117	286.235	SBR
315	0.93	14.41	55.6	71.7	16.1	45.768	63.905	74.953	92.464	117.955	SBR
316	0.98	20.85	60.1	75.0	16.1	47.262	67.341	78.448	91.797	114.177	SBR
317	0.92	24.99	52.1	71.7	13.6	23.072	31.424	36.736	46.897	56.974	SBR
318	0.91	29.33	48.1	71.0	14.4	16.934	22.921	26.949	35.522	43.373	SBR
319	0.73	9.99	40.4	58.6	-	42.374	61.444	71.279	111.286	150.369	SBR
320	0.72	14.82	31.8	56.9	-	27.089	37.644	43.018	67.843	86.585	SBR
321	0.68	19.66	38.7	56.1	-	20.149	28.051	32.068	51.991	65.373	SBR
322	0.73	24.41	36.8	60.4	-	14.760	20.133	23.191	37.631	46.886	SBR
323	0.70	29.58	35.9	55.5	-	12.736	17.281	20.008	32.560	40.124	SBR
202	1.17	25.51	57.6	72.5	14.2	45.746	65.789	88.121	82.007	99.447	PU
328	0.98	24.86	59.1	75.4	15.2	20.078	24.926	28.283	27.869	30.271	PU
330	0.99	14.89	57.2	74.5	14.1	39.565	48.013	53.480	53.717	57.546	PU
331	0.99	20.26	58.7	74.1	14.3	26.158	31.325	34.765	34.936	37.354	PU

**Table 2:** Static stiffness of SBR-based samples with a nominal density of 0.73 g/cm^3^.

Load Range [kN]	Static Stiffness *k_SP_* [MN/m]				
	319_SBR_10_0.73	320_SBR_15_0.73	321_SBR_20_0.73	322_SBR_25_0.73	323_SBR_30_0.73
16.0–40.8	41.998	27.104	20.303	14.850	12.902

**Table 3:** Static stiffness of PU-based samples with a nominal density of 1.00 g/cm^3^.

Load Range [kN]	Static Stiffness *k_SP_* [MN/m]		
	330_PU_15_1.00	330_PU_20_1.00	330_PU_25_1.00
16.0–40.8	39.668	26.116	20.049

**Table 4:** Dynamic stiffness at low frequencies of SBR-based samples with a nominal density of 0.73 g/cm^3^.

Frequency [Hz]	Dynamic Stiffness at Low Frequencies *k_LFPf_* [MN/m]				
	319_SBR_10_0.73	320_SBR_15_0.73	321_SBR_20_0.73	322_SBR_25_0.73	323_SBR_30_0.73
1	52.285	32.969	24.820	17.827	15.394
2	55.835	34.884	26.350	18.829	16.255
3	58.147	36.188	27.261	19.434	16.838
5	61.137	37.720	28.418	20.262	17.538
10	65.894	40.102	30.136	21.392	18.457
15	68.058	41.389	31.417	22.128	19.304
20	71.153	43.205	32.455	23.228	20.254

**Table 5:** Dynamic stiffness at high frequencies of SBR-based samples with a nominal density of 0.73 g/cm^3^.

Frequency [Hz]	Dynamic Stiffness at High Frequencies *k_HFPf_* [MN/m]				
	319_SBR_10_0.73	320_SBR_15_0.73	321_SBR_20_0.73	322_SBR_25_0.73	323_SBR_30_0.73
8	98.997	61.145	47.137	34.261	30.084
10	113.250	68.325	52.676	37.909	33.070
12.5	127.391	74.546	57.249	40.959	35.551
16	140.329	80.501	62.004	43.980	38.215
20	156.075	86.920	66.767	46.982	40.807
24	166.679	93.115	71.137	50.341	43.717

**Table 6:** Static stiffness of SBR- and PU-based samples with a nominal thickness of 25 mm.

Load Range [kN]	Static Stiffness *k_SP_* [MN/m]					
	307_SBR _25_1.10	312_SBR _25_1.05	317_SBR _25_0.95	322_SBR _25_0.73	328_PU _25_1.00	202_PU _25_1.15
16.0–40.8	243.137	68.367	22.685	14.850	20.049	46.704

**Table 7:** Subsets that determine the correlation strength [25].

Strong positive correlation	⟨0.7; 1.0)	
Moderate positive correlation	⟨0.4; 0.7)	
Weak positive correlation	⟨0; 0.4)	
Weak negative correlation	⟨−0.4; 0)	
Moderate negative correlation	⟨−0.7; −0.4)	
Strong negative correlation	⟨−1; −0.7)	

**Table 8:** Equivalent SBR- and PU-based materials.

No. (PU)	No. (SBR)	*h* (PU) [mm]	*h* (SBR) [mm]	Δ*h* [mm]	A (PU) [°ShA]	A (SBR) [°ShA]	*k_SP_* (PU) [MN/m]	*k_SP_* (SBR) [MN/m]	*k_LFPf_* 5 Hz (PU) [MN/m]	*k_LFPf_* 5 Hz (SBR) [MN/m]
202	315	25	15	10	57.6	55.6	45.746	45.768	65.789	63.905
328	317	25	25	0	59.1	52.1	20.078	23.072	24.926	31.424
330	313	15	30	15	57.2	60.8	39.565	41.905	48.013	60.586
331	317	20	25	−5	58.7	52.1	26.158	23.072	31.325	31.424

## Data Availability

The original contributions presented in the study are included in the article, and further inquiries can be directed to the corresponding author.
